# Improved understanding of biorisk for research involving microbial modification using annotated sequences of concern

**DOI:** 10.3389/fbioe.2023.1124100

**Published:** 2023-04-25

**Authors:** Gene D. Godbold, F. Curtis Hewitt, Anthony D. Kappell, Matthew B. Scholz, Stacy L. Agar, Todd J. Treangen, Krista L. Ternus, Jonas B. Sandbrink, Gregory D. Koblentz

**Affiliations:** ^1^ Signature Science, LLC, Charlottesville, VA, United States; ^2^ Signature Science, LLC, Austin, TX, United States; ^3^ Department of Computer Science, Rice University, Houston, TX, United States; ^4^ Nuffield Department of Medicine, University of Oxford, Oxford, United Kingdom; ^5^ Schar School of Policy and Government, George Mason University, Arlington, VA, United States

**Keywords:** microbial pathogenesis, DURC, functions of sequences of concern, FunSoCs, biothreat, biorisk, ontology

## Abstract

Regulation of research on microbes that cause disease in humans has historically been focused on taxonomic lists of ‘bad bugs’. However, given our increased knowledge of these pathogens through inexpensive genome sequencing, 5 decades of research in microbial pathogenesis, and the burgeoning capacity of synthetic biologists, the limitations of this approach are apparent. With heightened scientific and public attention focused on biosafety and biosecurity, and an ongoing review by US authorities of dual-use research oversight, this article proposes the incorporation of sequences of concern (SoCs) into the biorisk management regime governing genetic engineering of pathogens. SoCs enable pathogenesis in all microbes infecting hosts that are ‘of concern’ to human civilization. Here we review the functions of SoCs (FunSoCs) and discuss how they might bring clarity to potentially problematic research outcomes involving infectious agents. We believe that annotation of SoCs with FunSoCs has the potential to improve the likelihood that dual use research of concern is recognized by both scientists and regulators before it occurs.

## Introduction

In 2022, the National Institutes of Health (NIH) and Office of Science and Technology Policy (OSTP) began a process to evaluate the effectiveness of dual-use research oversight in the United States and determine whether the current approach sufficiently addresses future potential threats in biological research (Tabak and Jorgenson, 2022). This review encompasses three policies: the March 2012 United States Government Policy for Oversight of Life Sciences Dual Use Research of Concern (United States Government, 2012), the September 2014 United States Government Policy for Institutional Oversight of Life Sciences Dual Use Research of Concern (United States Government, 2014), and the December 2017 Framework for Guiding Funding Decisions about Proposed Research Involving Enhanced Potential Pandemic Pathogens (P3CO Framework) (Department of Health and Human Services, 2017). The March 2012 and September 2014 dual use research of concern (DURC) policies are complementary and will be considered together since they are both based on a shared list of pathogens and experiments that are subject to oversight. Under the DURC policies, research that is either conducted or funded by a federal agency on fifteen pathogens and toxins (Table 1) that is “reasonably anticipated” to produce one of seven experimental outcomes (Table 2) are subject to review by the funding agency. The list of pathogens is based on those deemed to be Tier 1 high-consequence biological threats by the Federal Select Agent Program (FSAP).

**TABLE 1:** Tier 1 pathogens of concern, federal select agent program.

Avian influenza virus (highly pathogenic)
*Bacillus anthracis*
Botulinum neurotoxin
*Burkholderia mallei*
*Burkholderia pseudomallei*
Ebola virus
Foot-and-mouth disease virus
*Francisella tularensis*
Marburg virus
Reconstructed 1918 Influenza virus
Rinderpest virus
Toxin-producing strains of *Clostridium botulinum*
Variola major virus
Variola minor virus
*Yersinia pestis*

**TABLE 2:** Dual-use research of concern (DURC).

a. Enhances the harmful consequences of the agent or toxin
b. Disrupts immunity or the effectiveness of an immunization against the agent or toxin without clinical or agricultural justification
c. Confers to the agent or toxin resistance to clinically or agriculturally useful prophylactic or therapeutic interventions against that agent or toxin or facilitates their ability to evade detection methodologies
d. Increases the stability, transmissibility, or the ability to disseminate the agent or toxin
e. Alters the host range or tropism of the agent or toxin
f. Enhances the susceptibility of a host population to the agent or toxin
g. Generates or reconstitutes an eradicated or extinct agent or toxin

The list-based approach of the DURC policies has been criticized for its static nature and lack of coverage of potentially risky research with pathogens that are not on the Select Agent Tier 1 list. A 2018 study by the National Academies of Science, Engineering, and Medicine highlighted the variety of ways in which biological threats beyond those on this specific list could be generated thanks to our improved understanding of which genotypes generate potentially harmful phenotypes and the diffusion of the expertise, techniques, and technologies needed to apply this knowledge to develop modified genomes with enhanced harmful attributes (National Academies of Science, Engineering, and Medicine, 2018).

The P3CO Framework provides for oversight of research funded by the Department of Health and Human Services (HHS) that is “reasonably anticipated” to enhance the lethality and/or transmissibility of a potential pandemic pathogen (PPP) which is a pathogen capable of “wide and uncontrollable spread” in human populations and able to cause “significant morbidity and/or mortality” in such a population. This type of research is known as “gain of function” since it results in a microbe with enhanced virulence, pathogenicity, transmissibility, or other attribute that poses a higher risk to the host population than the naturally occurring strain. Unlike the DURC policy, the P3CO Framework is not limited to a specified list of pathogens. However, both policies rely on an interpretation of which types of laboratory experiments can be “reasonably anticipated” to have the effects or outcomes covered by both policies. The Government Accountability Office (GAO) has highlighted the lack of a standard for judging what is “reasonably anticipated” as a weakness in the oversight of dual-use research (Government Accountability Office GAO, 2023).

Several of the more controversial “gain of function” experiments were the results of failures by scientists and/or funding agencies to “reasonably anticipate” the outcome of the proposed research. The canonical example is the insertion of the gene coding for the murine interleukin-4 (IL-4) immunomodulator into ectromelia virus (mousepox) by Australian scientists in 2001. This experiment resulted in a strain of the virus that was uniformly lethal to both susceptible and genetically resistant mice and, even more worryingly, killed 60% of mice vaccinated against the virus (Jackson et al., 2001). According to the authors of the study, “this came as a complete surprise and was totally unexpected.” However, it has been argued that previous work on IL-4 and poxviruses was such that the “available evidence fully predicted” that a recombinant mousepox IL-4 virus would be more virulent (Müllbacher and Lobigs, 2001), enhancing the harmful consequences of the agent [Table 2], disrupting host immunity [Table 2], and increasing the susceptibility of the host population to the virus [Table 2].

A more recent example also shows that potentially perilous engineering is not always identified in advance. In 2014, EcoHealth Alliance proposed a research project to the NIH to modify coronaviruses, including MERS and bat-related coronaviruses, to evaluate the pandemic risk they posed. NIH determined that these experiments did not fall under the scope of the P3CO Framework because the modifications were “not expected to generate viruses that would be more transmissible or more virulent in humans” despite this being the stated goal of the project. [Letter from NIH Director Francis Collins to Senator Charles Grassley, 28 July2021^1^.

The NIH was criticized when it became apparent that the resulting chimeric viruses were, in fact, more virulent in humanized animal models than the original strain^2^. In contrast, the Defense Advanced Research Projects Agency (DARPA) rejected a proposal from EcoHealth Alliance to fund similar research with chimeric coronaviruses using genes associated with the spike protein of SARS-related coronaviruses found in bats^3^.

Some countries use taxonomy-based lists for export controls, but only a handful of other countries use such lists to exercise oversight for dual-use research as the United States does. In the United Kingdom, the three largest funders of life sciences research—the Biotechnology and Biological Sciences Research Council, the Medical Research Council, and the Wellcome Trust—review research proposals for dual-use potential using the same list of experiments of concern as in the United States. However, the dual-use review process in the United Kingdom is applicable to all life sciences research, not just that conducted with a list of pathogens as in the United States^4^. Canada requires research institutions to develop plans for managing biorisks, including dual-use issues, and processes for scientists to report to their institution if their research could result in the creation of a “human pathogen with increased virulence, pathogenicity, or communicability, that is resistant to preventative or therapeutic treatments, or produces a toxin with increased toxicity”^5^. Australia does not have an explicit dual-use oversight policy, but research with infectious agents and creation of genetically modified organisms, including as a result of “gain of function” experiments, is subject to monitoring and reporting^6^.

## Sequences of concern (SoCs) as drivers of infectious diseases

We have been engaged in a multi-year effort to understand the risks of biological sequences and the sorts of threats they pose to humanity. Those that are likely to cause problems if moved to another organism have been called sequences of concern (National Research Council, 2010) which we abbreviate as SoCs. Techniques to transfer sequences from one microbe to another and alter them in ways both minor and major are widely available. We contend that SoCs are not confined to microbes that have historically been feared for their capacity for weaponization, but rather are found in all parasites that have evolved with specific host organisms to cause disease in those organisms. These are commonly called pathogens.

Thousands of published investigations in microbial pathogenesis have provided ample reason to think that the *direct* activity of SoCs on constituent molecules of the host is the primary driver of successful infection and pathogenesis. In the absence of these microbial sequences—which associate with and modify host molecules—infection cannot occur. There are also SoCs that act *indirectly*—by either altering molecules of the parasite or facilitating the operation of direct-acting SoCs (e.g., bacterial secretion system components and chaperones), but these are of secondary importance. Lastly there is the consideration of gene expression. If neither the direct- nor the indirect-acting SoCs of a bacterial or eukaryotic parasite are expressed in sufficient abundance or in a timely, coordinated manner, then the microbe will not be able to successfully exploit the host organism. We also recognize that there are transcriptional, translational, and post-translational influences. There can also be epigenetic effects modulating expression. We are not asserting that SoCs are the only contributors to pathogenic phenotypes, merely that, for microbial parasites, they are the essential contributors to such phenotypes for host organisms with normal immune systems and intact barriers. Without these sequences, the encoding microbes could not cause disease in the healthy, immune-normal hosts with which they co-evolved as pathogens.

In our earlier publication we described how we reviewed thousands of papers to find thousands of virulence factors from bacterial, viral, and eukaryotic parasites that were good candidates for SoCs. We pondered if a sequence, following transfer to another microbe, would be likely to enhance its ability to colonize a susceptible host, increasing the pathological consequences of infection. If the answer was ‘probably yes’, then we detailed its host-relevant activities and incorporated it in our dataset. For ∼100 sequences, the authors *demonstrated* the ability of the transferred sequence to exhibit the same or similar pathogenic function in a different microbe that was previously associated with the expression of that sequence in the original microbe (Godbold et al., 2022).

We developed a controlled vocabulary to describe SoCs called Functions of Sequences of Concern (FunSoCs) (Godbold et al., 2022). We used it for both machine learning and bioinformatic software (Balaji et al., 2022). With FunSoCs, we attempted to capture both the activity and the consequences of these sequences on the host during infection. We identified four types of host *damage* caused by SoCs: (1) cytotoxicity or cell membrane disruption, (2) tissue degradation, (3) organ disabling, and (4) inflammation. We also described types of *innate immune subversion* resulting from SoC activity including: (i) suppression of host immune signaling (with many subtypes), (ii) resisting phagocytosis, (iii) neutralizing host complement, (iv) countering antimicrobial peptide, (v) resisting oxidative killing, (vi) neutralizing host immunoglobulin, (vii) defeating host cytokine, and (viii) inhibiting antigen presentation. Two types of direct SoC activity are characteristic of nearly all infectious organisms: *adherence* and *invasion*. There are two functions of direct-acting SoCs peculiar to intracellular pathogens: *movement within a host cell* and *niche creation*. Finally, some SoCs provide pathogens the ability to *disseminate* within the host organism by subverting host barriers. In addition, we note which of nine areas of host cell biology (transcription, ubiquitination, etc.) are targeted by SoCs (Godbold et al., 2022).

A valuable adjunct to the consequentialist focus of FunSoCs is the pathogenesis gene ontology (PathGO) developed by researchers at the Johns Hopkins University Applied Physics Laboratory^7^. PathGO consists of ∼170 terms which are rooted in biological process and molecular function terms of the Gene Ontology resource (Ashburner et al., 2000; Gene Ontology Consortium et al., 2021). PathGO terms identify the host molecules and pathways that are the targets of SoC activity, and we have employed these to further specify SoCs in our dataset.

In the following sections we address the relative abundance of SoCs in pathogen genomes with reference to SARS-CoV-2 and *Bacillus anthracis*. We discuss whether some SoCs are worse or more dangerous than others with respect to their host-affecting properties and provide some examples of SoCs with multiple functions from bacterial, viral, and eukaryotic pathogens of humans. Next, we emphasize the importance of immune subverting SoCs as these sequences appear critical for producing host susceptibility to microbes. Then we consider the appropriate criteria for determining what microbes from which SoCs should be appropriated. In the final sections we grapple with how annotated SoCs can be used to guide biorisk management decisions. We provide a rubric (Table 6) that exemplifies how they might be applied to the USG dual-use research of concern policy to simplify decision-making processes. We close by drawing out implications of using SoCs to supplement the current taxonomic list-based approach for dual-use research oversight.

### How abundant are SoCs in pathogen genomes?

SoCs are more abundant in viral genomes as a fraction of the total genetic material than in other parasites. Of microbes capable of causing disease in humans, viruses are the most genetically compact. Even the largest of these (poxviruses) possess genomes two to three times smaller than that of the smallest bacterial pathogen (*Mycoplasma*). They contain, proportionally, more sequences that confound host immunity than bacterial, fungal, or protozoal parasites. Viruses abound in sequences disrupting innate immune signaling (Godbold et al., 2022). The larger viral pathogens for humans have DNA genomes and can allocate single sequences to one or just a few functions like soaking up host cytokines to blunt the local immune response (Dunlop et al., 2003; Seet et al., 2003; Alvarez-de Miranda et al., 2021). RNA viruses are necessarily more compact with each protein serving many functions. SARS-CoV-2 is an example. Of the ∼27 sequences that are translated into proteins (Jungreis et al., 2021), at least 24 are SoCs and 18 of those suppress host cellular immune defenses including Membrane (M), Nsp1, Nsp3, Nsp5, Nsp6, Nsp7, Nsp10, Nsp12, Nsp13, Nsp14, Nsp15, Nucleocapsid (N), Orf3a, Orf6, Orb7b, Orf8, Orf9b, and Spike (S). The hypervariable Orf8 is the only one of these that has so far been demonstrated to be dispensable (Zinzula, 2021). The immune subverting activity for the sequences that suppress host immunity are summarized in Table 3.

**TABLE 3:** SARS-CoV-2 encoded proteins directly involved in host immune subversion.

SoC	Innate immune subversion	FunSoCs	PathGO
Nsp1	Nsp1 shut down cellular translation, thereby abrogating much of the cellular innate immune defense (Thoms et al., 2020).	Manipulate host translation (Schubert et al., 2020; Thoms et al., 2020; Zhang et al., 2021a; Finkel et al., 2021); Suppress host immune signaling (Lei et al., 2020; Thoms et al., 2020; Shemesh et al., 2021);	PATHGO:0000006 (modulates protein synthesis in another organism) (Thoms et al., 2020); PATHGO:0000370 (mediates mRNA destruction in another organism) (Huang et al., 2011);
Nsp3	The protease Nsp3 cuts ISG15 from proteins to dampen inflammation and antiviral signaling (Klemm et al., 2020). It counteracted host antiviral ADP-ribosylation by poly-ADP-ribose polymerases (Rack et al., 2020; Russo et al., 2021), and also cleaved interferon response factor 3 (IRF3) (Moustaqil et al., 2021).	Manipulate host ubiquitin dynamics (Klemm et al., 2020); Suppress host immune signaling (Klemm et al., 2020; Lei et al., 2020; Rack et al., 2020; Shin et al., 2020; Moustaqil et al., 2021; Correy et al., 2022); Resist other immune effector (Rack et al., 2020; Brosey et al., 2021; Schuller et al., 2021); Degrade tissue (cytopathic effect) (Shin et al., 2020);	PATHGO:0000382 (suppresses interferon signaling in another organism) (Lei et al., 2020; Moustaqil et al., 2021); PATHGO:0000325 (modulates ubiquitin dynamics in another organism) (Klemm et al., 2020); PATHGO:0000330 (mediates de-ISGylation of proteins in another organism) (Klemm et al., 2020); PATHGO:0000365 (mediates de-ADP-ribosylation of proteins in another organism) (Rack et al., 2020);
Nsp5	The Nsp5 protease cut human TAB1, the intracellular pattern recognition receptor NLRP12 (Moustaqil et al., 2021), and human gasdermin (Shi et al., 2022). It promoted the ubiquitination and subsequent destruction of host MAVS. Nsp5 cut the N-terminus of RIG-1 to eliminate its ability to trigger downstream interferon production (Liu et al., 2021). Nsp5 disrupted formation of cellular stress granules and the consequent interaction of RIG-1 and MAVS (Zheng et al., 2022).	Manipulate host ubiquitin dynamics (Liu et al., 2021); Suppress host immune signaling (Liu et al., 2021; Shemesh et al., 2021);	PATHGO:0000325 (modulates ubiquitin dynamics in another organism) (Liu et al., 2021); PATHGO:0000306 (disrupts RIG-I signaling in another organism) (Liu et al., 2021);
Nsp6	Nsp6 associated with TANK-binding kinase 1 to suppress IRF3 phosphorylation and subsequent interferon-beta production (Xia et al., 2020; Vazquez et al., 2021).	Manipulate host membrane dynamics (Díaz, 2020; Mishra et al., 2021); Suppress host immune signaling (Xia et al., 2020; Shemesh et al., 2021);	PATHGO:0000382 (suppresses interferon signaling in another organism) (Xia et al., 2020); PATHGO:0000236 (modulates cell endomembrane dynamics in another organism) (Díaz, 2020);
Nsp12	Nsp12 inhibited IFN promoter activation triggered by overexpression of RIG-I, MDA5, MAVS, and IRF3. This suppression was not dependent upon the polymerase activity of Nsp12 (Wang et al., 2021c).	Suppress host immune signaling (Lei et al., 2020; Wang et al., 2021c);	PATHGO:0000382 (suppresses interferon signaling in another organism) (Wang et al., 2021c);
Nsp13	Nsp13 associated with TANK-binding kinase 1 to suppress IRF3 phosphorylation and subsequent interferon-beta production (Xia et al., 2020; Vazquez et al., 2021). Nsp13 associated with STAT1 to suppress interferon signaling (Feng et al., 2021).	Manipulate host ubiquitin dynamics (Guo et al., 2021); Manipulate host membrane dynamics (Díaz, 2020; Gordon et al., 2020); Suppress host immune signaling (Lei et al., 2020; Xia et al., 2020; Zhang et al., 2021b; Feng et al., 2021);	PATHGO:0000382 (suppresses interferon signaling in another organism) (Xia et al., 2020); PATHGO:0000236 (modulates cell endomembrane dynamics in another organism) (Díaz, 2020); PATHGO:0000325 (modulates ubiquitin dynamics in another organism) (Guo et al., 2021); PATHGO:0000302 (disrupts JAK-STAT signaling in another organism) (Feng et al., 2021);
Nsp14	The NSP14 exonuclease antagonized host cell interferon production and host IRF3 nuclear translocation (Yuen et al., 2020). Nsp14 mediates the cessation of host cell translation. Mutations in the active site of either abolish its ability to inhibit translation. Nsp14 forms a complex with Nsp10 that enhances its ability to inhibit translation and so abolishes the induction of immune evasion genes by interferon (Hsu et al., 2021).	Manipulate host translation (Hsu et al., 2021); Suppress host immune signaling (Lei et al., 2020; Yuen et al., 2020; Hsu et al., 2021);	PATHGO:0000327 (mediates DNA cleavage in another organism) (Yuen et al., 2020); PATHGO:0000382 (suppresses interferon signaling in another organism) (Yuen et al., 2020); PATHGO:0000006 (modulates protein synthesis in another organism) (Hsu et al., 2021);
Nsp15	Nsp15 interfered with IFN-alpha/beta production through its interaction with the host E3 ligase RNF41/Nrdp1 (Gordon et al., 2020).	Manipulate host ubiquitin dynamics (Gordon et al., 2020); Manipulate host membrane dynamics (Díaz, 2020); Suppress host immune signaling (Yuen et al., 2020; Shemesh et al., 2021);	PATHGO:0000236 (modulates cell endomembrane dynamics in another organism) (Díaz, 2020); PATHGO:0000306 (disrupts RIG-I signaling in another organism) (Shemesh et al., 2021); PATHGO:0000325 (modulates ubiquitin dynamics in another organism) (Gordon et al., 2020);
Orf3a	Orf3a upregulated suppressor of cytokine signaling (SOCS1) to inhibit antiviral JAK/STAT signaling (Wang et al., 2021a). It is associated with the host E3 ubiquitin ligase TRIM59 which regulates antiviral immune signaling (Gordon et al., 2020).	Manipulate host transcription (Wang et al., 2021a); Manipulate host ubiquitin dynamics (Gordon et al., 2020); Manipulate host regulated cell death (Ren et al., 2020); Manipulate host membrane dynamics (Chen et al., 2021); Manipulate xenophagy (Chen et al., 2021; Miao et al., 2021; Su et al., 2021; Zhang et al., 2022); Suppress host immune signaling (Gordon et al., 2020; Wang et al., 2021a);	PATHGO:0000335 (induces apoptosis in another organism) (Ren et al., 2020); PATHGO:0000239 (disrupts phagolysosome fusion in another organism) (Zhang et al., 2021d; Miao et al., 2021); PATHGO:0000347 (modulates autophagy or xenophagy in another organism) (Zhang et al., 2021d; Miao et al., 2021); PATHGO:0000302 (disrupts JAK-STAT signaling in another organism) (Wang et al., 2021a); PATHGO:0000326 (modulates transcription in another organism) (Wang et al., 2021a);
Orf6	Orf6 associated with importin karyopherin-alpha2 (KPNA2) to inhibit translocation of IRF3 to the nucleus (Xia et al., 2020). The C-terminus of Orf6 directly binds to STAT1 resulting in its exclusion from the host nucleus (Miyamoto et al., 2022).	Manipulate host translation (Gordon et al., 2020; Addetia et al., 2021); Suppress host immune signaling (Lei et al., 2020; Li et al., 2020, 8; Xia et al., 2020; Yuen et al., 2020; Miyamoto et al., 2022);	PATHGO:0000006 (modulates protein synthesis in another organism) (Gordon et al., 2020); PATHGO:0000382 (suppresses interferon signaling in another organism) (Xia et al., 2020);
Orf8	Orf8 mediated immune evasion via downregulation of host MHC-I (Flower et al., 2020; Park, 2020). MHC-I molecules are targeted for lysosomal destruction by autophagy through the host beclin-1-mediated pathway (Zhang et al., 2021c).	Manipulate host membrane dynamics (Díaz, 2020); Suppress host immune signaling (Li et al., 2020, 8); Inhibit host antigen presentation (Flower et al., 2020, 8; Park, 2020, 8; Zhang et al., 2021c; Matsuoka et al., 2022); Induce inflammation (Lin et al., 2021; Zinzula, 2021);	PATHGO:0000308 (disrupts antigen presentation in another organism) (Flower et al., 2020, 8; Park, 2020, 8); PATHGO:0000351 (mediates cytokine sequestration in another organism) (Lin et al., 2021); PATHGO:0000236 (modulates cell endomembrane dynamics in another organism) (Díaz, 2020); PATHGO:0000362 (suppresses anti-inflammatory cytokine activity in another organism) (Lin et al., 2021);
Orf9b	Orf9b localized to the membrane of host mitochondria and suppressed host type I interferon (IFN) responses by targeting host TOM70 (Jiang et al., 2020; Brandherm et al., 2021). Orf9b antagonized the cellular antiviral response by targeting the NFκB essential modulator (NEMO, IKKγ). This association disrupted the polyubiquitination of NEMO and inhibited NFκB signaling (Wu et al., 2021).	Manipulate host ubiquitin dynamics (Wu et al., 2021); Suppress host immune signaling (Kreimendahl and Rassow, 2020; Han et al., 2021; Wu et al., 2021);	PATHGO:0000306 (disrupts RIG-I signaling in another organism) (Kreimendahl and Rassow, 2020); PATHGO:0000325 (modulates ubiquitin dynamics in another organism) (Wu et al., 2021); PATHGO:0000295 (suppresses NFκB signaling in another organism) (Wu et al., 2021); PATHGO:0000300 (suppresses STING signaling in another organism) (Han et al., 2021); PATHGO:0000352 (disrupts TRIM/TRIM-like signaling in another organism) (Han et al., 2021); PATHGO:0000382 (suppresses interferon signaling in another organism) (Han et al., 2021);
M	M localizes to the host ER and Golgi and colocalizes with host TBK1 and TRAF3 but just partially with RIG-I, MDA-5, and MAVS. Membrane prevents the interaction of RIG-I with MAVS, MAVS with TBK1, and TRAF3 with TBK1. IRF3 phosphorylation is inhibited (Zheng et al., 2020). Membrane protein suppresses expression of IFNβ and interferon-stimulated genes by interacting with MDA5, TRAF3, IKKε, and TBK1. Membrane protein induces the degradation of TBK1 by Lys48-linked ubiquitination. Lower levels of TBK1 impair formation of the TRAF3-TANK-TBK1/IKKε complex leading to inhibition of IFN-I (Sui et al., 2021).	Manipulate host ubiquitin dynamics (Sui et al., 2021, 1); Manipulate host regulated cell death (Yang et al., 2022); Manipulate host membrane dynamics (Díaz, 2020); Suppress host immune signaling (Lei et al., 2020; Zheng et al., 2020; Sui et al., 2021, 1); Resist other host immune effector (Zhang et al., 2021b);	PATHGO:0000325 (modulates ubiquitin dynamics in another organism) (Sui et al., 2021, 1); PATHGO:0000236 (modulates cell endomembrane dynamics in another organism) (Díaz, 2020); PATHGO:0000382 (suppresses interferon signaling in another organism) (Lei et al., 2020); PATHGO:0000306 (disrupts RIG-I signaling in another organism) (Fu et al., 2021; Sui et al., 2021, 1); PATHGO:0000314 (modulates TRAF signaling in another organism) (Sui et al., 2021, 1);
N	Nucleocapsid suppressed the interaction between the host TRIM25 proteins and RIG-I (Oh and Shin, 2021). It also interacted with both STAT1 and STAT2 to suppress their nuclear translocation (Mu et al., 2020). Nucleocapsid bound host G3BP1 and thereby contributed to the dispersion of host stress granules where antiviral signaling is facilitated (Biswal et al., 2022; Zheng et al., 2022).	Manipulate host translation (Gordon et al., 2020; Lu et al., 2021); Adherence to another organism (Kumar et al., 2020); Suppress host immune signaling (Li et al., 2020, 8; Mu et al., 2020; Oh and Shin, 2021; Wang et al., 2021b; Zheng et al., 2022); Induce inflammation (Kumar et al., 2020; Magro et al., 2020; Youn et al., 2021);	PATHGO:0000006 (modulates protein synthesis in another organism) (Gordon et al., 2020); PATHGO:0000306 (disrupts host RIG-I signaling) (Mu et al., 2020); PATHGO:0000302 (disrupts JAK-STAT signaling in another organism) (Mu et al., 2020); PATHGO:0000382 (suppresses interferon signaling in another organism) (Mu et al., 2020); PATHGO:0000361 (enhances coagulation in another organism) (Magro et al., 2020; Youn et al., 2021); PATHGO:0000352 (disrupts TRIM/TRIM-like signaling in another organism) (Oh and Shin, 2021); PATHGO:0000072 (mediates binding to cell surface glycoprotein in another organism) (Kumar et al., 2020);
S	The S1 portion of spike directly interacted with STAT1 to interfere with the interaction between JAK1 and STAT1 and suppressed STAT1 phosphorylation (Zhang et al., 2021b).	Manipulate host membrane dynamics (Prelli Bozzo et al., 2021); Adherence to another organism (Cantuti-Castelvetri et al., 2020; Daly et al., 2020; Saputri et al., 2020); Host invasion (Cantuti-Castelvetri et al., 2020; Daly et al., 2020; Walls et al., 2020); Suppress host immune signaling (Zhang et al., 2021b); Induce inflammation (Cao et al., 2020; Barreda et al., 2021; Khan et al., 2021; Shirato and Kizaki, 2021; Youn et al., 2021); Degrade tissue (Buchrieser et al., 2020; Barrett et al., 2021; Rocheleau et al., 2021; Yu et al., 2022);	PATHGO:0000072 (mediates binding to cell surface glycoprotein in another organism) (Saputri et al., 2020); PATHGO:0000368 (mediates host cell invasion by microbe) (Walls et al., 2020); PATHGO:0000003 (modulates ion channel activity in another organism) (Braga et al., 2021, 16); PATHGO:0000358 (mediates release of cell from extracellular matrix in another organism) (Braga et al., 2021, 16); PATHGO:0000162 (disrupts epithelial layer in another organism) (Braga et al., 2021, 16); PATHGO:0000302 (disrupts JAK-STAT signaling in another organism) (Zhang et al., 2021b);

Our annotations suggest that, in contrast to viruses, the great majority of the encoded sequences of *nonviral* microbes play no role in pathogenesis. These SoCs comprise, at most, a few per cent of the sequences of bacterial, fungal, and protozoal pathogen genomes. Some microbes do not have nearly so many. Out of ∼5,800 genes on a single chromosome and two plasmids, *Bacillus anthracis* encodes about a dozen proteins enabling pathogenesis including the ‘big three’ of protective antigen, edema factor, and lethal factor. The annotated proteins are shown in Table 4.

**TABLE 4:** Sequences of concern of *Bacillus anthracis*.

Sequence of Concern	Function of Sequences of Concern (FunSoCs)	Pathogenesis Gene Ontology (PathGO)
Adenosine synthase A	Suppress host immune signaling (Thammavongsa et al., 2009)	PATHGO:0000220 (suppresses inflammatory cytokine release in another organism) (Thammavongsa et al., 2009)
Anthrolysin O	Adherence to another organism (Mosser and Rest, 2006); Dissemination in host (Bishop et al., 2010); Resist other immune effector (Mosser and Rest, 2006; Heffernan et al., 2007);	PATHGO:0000211 (mediates binding to the cell surface in another organism) (Mosser and Rest, 2006); PATHGO:0000033 (mediates pore formation in another organism) (Mosser and Rest, 2006); PATHGO:0000253 (mediates barrier traversal in another organism) (Bishop et al., 2010);
BclA	Resist host complement (Wang et al., 2016a);	PATHGO:0000341 (mediates binding of complement control protein in another organism) (Wang et al., 2016a);
BslA	Adherence to another organism (Ebrahimi et al., 2009; Kern and Schneewind, 2010; Wang et al., 2016b); Dissemination in host (Ebrahimi et al., 2009);	PATHGO:0000275 (mediates binding to laminin in another organism) (Wang et al., 2016b); PATHGO:0000253 (mediates barrier traversal in another organism) (Ebrahimi et al., 2009); PATHGO:0000211 (mediates binding to the cell surface in another organism) (Ebrahimi et al., 2009);
ClpX	Resist host antimicrobial peptide (McGillivray et al., 2009);	PATHGO:0000104 (disrupts antimicrobial peptide binding in another organism) (McGillivray et al., 2009);
Immune inhibitor A	Dissemination in host (Mukherjee et al., 2011; Tonry et al., 2012);	PATHGO:0000253 (mediates barrier traversal in another organism) (Mukherjee et al., 2011; Tonry et al., 2012);
PI-PLC	Resist other immune effector (Wei et al., 2005; Zenewicz et al., 2005);	PATHGO:0000233 (disrupts toll-like receptor signaling in another organism) (Zenewicz et al., 2005); PATHGO:0000080 (suppresses dendritic cell activation in another organism) (Zenewicz et al., 2005); PATHGO:0000055 (mediates membrane phospholipid cleavage in another organism) (Zenewicz et al., 2005);
Superoxide dismutases (4)	Resist host oxidative killing (Cybulski et al., 2009)	PATHGO:0000230 (mediates free radical detoxification) (Cybulski et al., 2009); PATHGO:0000271 (mediates resistance to oxidative killing in another organism) (Cybulski et al., 2009);
Protective antigen	Adherence to another organism (Vuyisich et al., 2012); Host invasion (Abrami et al., 2005);	PATHGO:0000072 (mediates binding to cell surface glycoprotein in another organism) (Vuyisich et al., 2012); PATHGO:0000369 (mediates cell invasion by macromolecule from another organism) (Abrami et al., 2005); PATHGO:0000033 (mediates pore formation in another organism) (Abrami et al., 2005);
Edema factor	Suppress host immune signaling (Agrawal and Pulendran, 2004; Tournier et al., 2005; van Sorge et al., 2008); Disable organ (Firoved et al., 2005; Guichard et al., 2010; Liu et al., 2013; Hutt et al., 2014)	PATHGO:0000173 (modulates cAMP synthesis within a cell of another organism) (Agrawal and Pulendran, 2004; Friebe et al., 2016); PATHGO:0000220 (suppresses inflammatory cytokine release in another organism) (Tournier et al., 2005; van Sorge et al., 2008); PATHGO:0000080 (suppresses dendritic cell activation in another organism) (Tournier et al., 2005); PATHGO:0000326 (modulates transcription in host cell) (van Sorge et al., 2008);
Lethal factor	Adherence to another organism (Vuyisich et al., 2012); Dissemination in host (Langer et al., 2012); Suppress host immune signaling (Agrawal et al., 2003; Tournier et al., 2005; van Sorge et al., 2008; Friebe et al., 2016; Goldberg et al., 2017); Induce inflammation (Chui et al., 2019); Degrade tissue (Langer et al., 2012); Disable organ (Guichard et al., 2010; Liu et al., 2013; Hutt et al., 2014);	PATHGO:0000211 (mediates binding to the cell surface in another organism) (Vuyisich et al., 2012); PATHGO:0000220 (suppresses inflammatory cytokine release in another organism) (van Sorge et al., 2008; Friebe et al., 2016); PATHGO:0000290 (suppresses MAPK signaling in another organism) (Friebe et al., 2016); PATHGO:0000080 (suppresses dendritic cell activation in another organism) (Agrawal et al., 2003; Tournier et al., 2005); PATHGO:0000349 (enhances inflammasome activation in another organism) (Chui et al., 2019); PATHGO:0000162 (disrupts epithelial layer in another organism) (Langer et al., 2012); PATHGO:0000253 (mediates barrier traversal in another organism) (Langer et al., 2012);

### Are some SoCs ‘worse’ than others?

We think the following assertions are generally true about the relative danger of SoCs in microbial pathogenesis. First, SoCs that act directly on a host molecule are more concerning than those that act indirectly. Second, SoCs that have a *damaging* effect are more concerning than those that only provide adhesive, invasive, or disseminating capacities. We think that SoCs enabling dissemination of a pathogen that has already colonized a host are more concerning than adhesive or invasive SoCs. Third, SoCs that only provide within-cell motility or the ability to form an intracellular niche are the SoCs of lowest concern of the direct-acting SoCs. Fourth, SoCs that have multiple functions are more concerning than those that have a single function. Some sequences that enable adhesion can also subvert immunity. A subset of SoCs with many functions are detailed in Table 5. *Immune subverting* SoCs are a special case that we address in the next section.

**TABLE 5:** SoCs with multiple functions from bacterial, viral, and eukaryotic pathogens.

SoC, Organism	FunSoCs	PathGO terms
LasB, *Pseudomonas aeruginosa*	Resist host complement (Bastaert et al., 2018); Resist host antimicrobial peptide (Saint-Criq et al., 2018); Resist host oxidative killing (Bastaert et al., 2018); Counter host cytokine (Matheson et al., 2006; Golovkine et al., 2014); Resist other host immune effector (Ijiri et al., 1994); Induce inflammation (Saint-Criq et al., 2018; Sun et al., 2020); Degrade tissue (Leduc et al., 2007; Beaufort et al., 2011; Golovkine et al., 2014); Disable organ (Zhu et al., 2021);	PATHGO:0000271 (mediates resistance to oxidative killing in another organism) (Bastaert et al., 2018); PATHGO:0000353 (modulates reactive oxygen species levels in another organism) (Bastaert et al., 2018); PATHGO:0000100 (mediates resistance to complement system in another organism) (Bastaert et al., 2018); PATHGO:0000104 (disrupts antimicrobial peptide binding in another organism) (Saint-Criq et al., 2018); PATHGO:0000363 (suppresses pro-inflammatory cytokine activity in another organism) (Matheson et al., 2006); PATHGO:0000214 (modifies tight junction or adherens junction in another organism) (Golovkine et al., 2014); PATHGO:0000358 (mediates release of cell from extracellular matrix in another organism) (Leduc et al., 2007);
IbpA, *Histophilus somni*	Manipulate host small GTPase (Zekarias et al., 2010); Manipulate host cytoskeleton dynamics (Zekarias et al., 2010); Adherence to another organism (Zekarias et al., 2010; Corbeil, 2016); Resist host phagocytosis (Pan et al., 2018); Resist host complement (Pan et al., 2018); Counter host immunoglobulin (Corbeil, 2016); Cytotoxicity (Zekarias et al., 2010);	PATHGO:0000355 (mediates deactivation of small GTPase in another organism) (Zekarias et al., 2010); PATHGO:0000216 (mediates filamentous actin depolymerization in another organism) (Zekarias et al., 2010); PATHGO:0000211 (mediates binding to the cell surface in another organism) (Corbeil, 2016); PATHGO:0000232 (suppresses phagocytosis in another organism) (Pan et al., 2018); PATHGO:0000257 (mediates immunoglobulin neutralization in another organism) (Corbeil, 2016); PATHGO:0000100 (mediates resistance to complement system in another organism) (Pan et al., 2018);
IpaB, *Shigella flexneri*	Manipulate host cell cycle (Iwai et al., 2007; Wang et al., 2019, 7); Secretion system component (Blocker et al., 1999; Iwai et al., 2007; Roehrich et al., 2010); Adherence to another organism (Schroeder and Hilbi, 2008); Host invasion (Lafont et al., 2002; Mounier et al., 2009; 2012; Senerovic et al., 2012); Suppress host immune signaling (Hathaway et al., 2002); Induce inflammation (Hilbi et al., 1998; Senerovic et al., 2012); Cytotoxicity (Yang et al., 2015);	PATHGO:0000152 (induces cell cycle arrest in cell of another organism) (Iwai et al., 2007); PATHGO:0000110 (mediates secretion of protein effector) (Blocker et al., 1999; Roehrich et al., 2010); PATHGO:0000234 (mediates binding to integrin in another organism) (Schroeder and Hilbi, 2008); PATHGO:0000368 (mediates host cell invasion by microbe) (Lafont et al., 2002); PATHGO:0000220 (suppresses inflammatory cytokine release in another organism) (Hathaway et al., 2002); PATHGO:0000284 (mediates binding to cholesterol in another organism) (Mounier et al., 2012); PATHGO:0000033 (mediates pore formation in another organism) (Mounier et al., 2009; Senerovic et al., 2012);
TcdA, *Clostridioides difficile*	Manipulate host small GTPase (Aktories et al., 2017); Manipulate host cytoskeleton dynamics (Aktories et al., 2017); Adherence to another organism (Aktories and Just, 2005; Tao et al., 2019); Host invasion (Papatheodorou et al., 2010; Aktories et al., 2017); Induce inflammation (Ng et al., 2010; Cowardin et al., 2016); Degrade tissue (Aktories et al., 2017);	PATHGO:0000285 (mediates carbohydrate-derivative binding in another organism) (Aktories and Just, 2005); PATHGO:0000273 (mediates glycosaminoglycan- or proteoglycan-binding in another organism) (Tao et al., 2019); PATHGO:0000072 (mediates binding to cell surface glycoprotein in another organism) (Tao et al., 2019); PATHGO:0000214 (modifies tight junction or adherens junction in another organism) (Sousa et al., 2005); PATHGO:0000369 (mediates cell invasion by macromolecule from another organism) (Papatheodorou et al., 2010); PATHGO:0000355 (mediates deactivation of small GTPase in another organism) (Aktories et al., 2017); PATHGO:0000214 (modifies tight junction or adherens junction in another organism) (Aktories et al., 2017); PATHGO:0000216 (mediates filamentous actin depolymerization in another organism) (Aktories et al., 2017); PATHGO:0000162 (disrupts epithelium in another organism) (Aktories et al., 2017);
NS1, influenza virus	Manipulate host transcription (Anastasina et al., 2016); Manipulate host translation (Chaimayo et al., 2018); Manipulate host ubiquitin dynamics (Gack et al., 2009); Manipulate host regulated cell death (Bergsbaken et al., 2009); Suppress host immune signaling (Fislová and Kostolanský, 2005; Gack et al., 2009); Resist other host immune effector (Fernandez-Sesma et al., 2006); Suppress antigen presentation (Chien et al., 2004; Bonjardim, 2005);	PATHGO:0000326 (modulates transcription in another organism) (Anastasina et al., 2016); PATHGO:0000006 (modulates protein synthesis in another organism) (Chaimayo et al., 2018); PATHGO:0000325 (modulates ubiquitin dynamics in another organism) (Gack et al., 2009); PATHGO:0000352 (disrupts TRIM/TRIM-like signaling in another organism) (Gack et al., 2009); PATHGO:0000334 (suppresses apoptosis in another organism) (Bergsbaken et al., 2009); PATHGO:0000220 (suppresses inflammatory cytokine release in another organism) (Fislová and Kostolanský, 2005); PATHGO:0000080 (suppresses dendritic cell activation in another organism) (Fernandez-Sesma et al., 2006); PATHGO:0000312 (mediates concealment of foreign nucleic acid in another organism) (Chien et al., 2004; Bonjardim, 2005);
E1A, human adenovirus	Manipulate host transcription (Fonseca et al., 2012; Glenewinkel et al., 2016; King et al., 2018); Manipulate host cell cycle (Ryan, 2010); Manipulate host ubiquitin dynamics (Fonseca et al., 2012); Manipulate host regulated cell death (Miller, 2005); Suppress host immune signaling (Lau et al., 2015); Suppress antigen presentation (Jiao et al., 2010; Berhane et al., 2011)	PATHGO:0000325 (modulates ubiquitin dynamics in another organism) (Fonseca et al., 2012); PATHGO:0000326 (modulates transcription in another organism) (Fonseca et al., 2012; Glenewinkel et al., 2016; King et al., 2018); PATHGO:0000335 (induces apoptosis in another organism) (Miller, 2005); PATHGO:0000300 (disrupts STING signaling in another organism) (Lau et al., 2015); PATHGO:0000308 (disrupts antigen presentation in another organism) (Jiao et al., 2010; Berhane et al., 2011);
NSs, Rift Valley fever virus	Manipulate host transcription (Kainulainen et al., 2014; Terasaki et al., 2016); Manipulate host cell cycle (Baer et al., 2012); Manipulate host ubiquitin dynamics (Kainulainen et al., 2014; 2016); Manipulate host cytoskeleton dynamics (Bamia et al., 2020); Suppress host immune signaling (Le May et al., 2008; Head et al., 2012; Terasaki et al., 2016); Resist other host immune effector (Kainulainen et al., 2016; Terasaki et al., 2016);	PATHGO:0000326 (modulates transcription in another organism) (Kainulainen et al., 2014; Terasaki et al., 2016); PATHGO:0000152 (induces cell cycle arrest in cell of another organism) (Baer et al., 2012); PATHGO:0000325 (modulates ubiquitin dynamics in another organism) (Kainulainen et al., 2014; 2016); PATHGO:0000028 (modulates cytoskeleton in another organism) (Bamia et al., 2020); PATHGO:0000214 (modifies tight junction or adherens junction in another organism) (Bamia et al., 2020); PATHGO:0000382 (suppresses interferon signaling in another organism) (Le May et al., 2008; Head et al., 2012); PATHGO:0000304 (disrupts PKR activity in another organism) (Kainulainen et al., 2016; Terasaki et al., 2016);
Alp1, *Neosartorya fumigata*	Resists host complement (Behnsen et al., 2010); Counter host immunoglobulin (Behnsen et al., 2010); Degrade tissue (Balenga et al., 2015); Disable organ (Balenga et al., 2015);	PATHGO:0000100 (mediates resistance to complement system in another organism) (Behnsen et al., 2010); PATHGO:0000257 (mediates immunoglobulin neutralization in another organism) (Behnsen et al., 2010); PATHGO:0000226 (disrupts extracellular matrix in another organism) (Balenga et al., 2015);
ROP18/VIR3, *Toxoplasma gondii*	Manipulate host ubiquitin dynamics (Du et al., 2014); Manipulate host programmed cell death (Wu et al., 2016); Suppress host immune signaling (Fentress et al., 2010; Yamamoto et al., 2011; Du et al., 2014; Yang et al., 2017; Xia et al., 2018; Yao et al., 2021)	PATHGO:0000325 (modulates ubiquitin dynamics in another organism) (Du et al., 2014); PATHGO:0000295 (suppresses NFκB signaling in another organism) (Du et al., 2014); PATHGO:0000334 (suppresses apoptosis in another organism) (Wu et al., 2016); PATHGO:0000352 (disrupts TRIM/TRIM-like signaling in another organism) (Yao et al., 2021);

### The importance of immune subverting SoCs for host susceptibility

Why are some organisms susceptible to infection by some microbes but not others? Why are immune-compromised persons subject to infection with a broader range of parasites than immune-normal persons? Why do defects in immune detectors and immune effectors of an organism allow microbes that are normally incapable of infection to become competent for infection and pathogenesis? A single amino acid change in an immune effector can mean the difference between life and death during challenge with a virus (Andoniou et al., 2014). The study of human immune deficiencies shows the critical importance of components of innate immunity for defense against the specific, usually narrow, set of parasites against which they defend (Casanova, 2015a; 2015b; Li et al., 2017).

What these phenomena have in common is a host with intact barriers and an immune system that fends off microbes that lack direct-acting sequences evolved to either counter or disrupt key components of the innate immune system of that host (Godbold et al., 2022). These direct-acting sequences, expressed in a combination that varies by parasitic microbe, produce a state of susceptibility in a host, allowing colonization by the parasite (Wickham et al., 2007; Kurupati et al., 2010). Such a set of immune subverting mechanisms is not generic. A parasite with a given set of innate immune subverting mechanisms is not able to subvert every immune system, but just the limited grouping of species with which it co-evolved as a pathogen. Its encoded molecular armamentarium is specific to counter a relatively narrow set of organism-specific innate signaling pathways and effectors and exploit a specific host biology—including barrier breaching. These encoded sequences are how the pathogen makes a host susceptible. Obviously jumps into new species can happen. In these cases, though, the new species, if it is not immune compromised, is always related to the original species with respect to the innate immune system. A mouse pathogen innate immune subverting mechanisms may (or may not) function on the human ortholog of the mouse innate immune protein. But the sequences encoded by a microbe that make plants susceptible to that particular pathogen by subverting the plant innate immune defenses do not, and cannot, make mammals susceptible to that same microbe because of the substantial differences in the innate immune systems of plants and mammals. *Yersinia pestis* infects two different sorts of hosts: insects (fleas) and mammals. The bacterium encodes different sets of sequences to exploit each host and employs temperature-based regulation to switch between them (Vadyvaloo et al., 2010).

We are asserting that the immune subverting mechanisms employed by a specific microbial pathogen are what produce susceptibility in the (typically) narrow range of hosts parasitized by that microbe. Whatever pathogenesis follows from this infection is not just a function of the parasite, but rather an emergent property of the gestalt of the host-parasite interactions shaped by the development of the adaptive immune response (Casadevall and Pirofski, 2003; Pirofski and Casadevall, 2015). For these reasons, we think it is possible that immune subverting mechanisms may be the ‘worst’ of SoCs since they essentially enable infection and appear (to us) to be the difference between pathogenic and non-pathogenic species. We have documented and annotated a few thousand SoCs from parasites of (mostly) humans, including over 500 that subvert host innate immunity. But this is probably just a tithe of the immune subverting SoCs encoded by human pathogens. While we think that the available evidence points strongly in the direction of these sequences being necessary for infection by making the host susceptible, at present this is merely a hypothesis that requires testing.

### From how broad a pool of pathogens should SoCs be drawn? Which hosts?

Every nonviral species in biology serves as a host to its own subset of microbial pathogens. And all those pathogens have sequences that directly exploit the biology of their host. But these are not necessarily SoCs. Why not? Because sequences of concern are only ‘concerning’ if they are from pathogens capable of infecting humans and other species that humans rely upon for survival. The specific bacteriophage sequences enabling exploitation of strains of *Salmonella* or *Listeria* are not SoCs because humans do not care about the wellbeing of those bacteria. The sequences that allow bacteriophage to exploit these bacteria cannot be used to cause harm to mammalian or crop plant hosts and so would not be considered SoCs. Sequences encoding virulence factors that are designated SoCs should be documented and annotated only from pathogens that afflict humans, our livestock, and our crop plants (Godbold et al., 2022).

As mentioned above, this requires a broadening of microbes beyond those placed on select agent lists. These lists are generally composed of organisms and toxins that have been weaponized or are viewed as being weaponizable. But sequences that effectively interact with human molecules, attach to host cells, invade them, subvert immunity, enable dissemination, and generate pathology are not limited to weaponizable microbes. If microbes that cause disease in immune-compromised people are included, there are at least 1,500 species that probably encode SoCs (Godbold et al., 2022).

That said, we are not sure where the line on infectious microbes should be drawn: all microbes capable of causing disease in any human, however immune-compromised? That would require mining SoCs from opportunistic pathogens. Or should SoCs be taken only from microbes capable of causing disease in immune-normal people? The latter would neglect documenting the many and varied SoCs that have been elegantly investigated in such ‘conditional’ pathogens as *Pseudomonas aeruginosa*.

We mention SoCs from pathogens of crop plants above, but we acknowledge that is the weakest area of our SoC annotation effort. This is the case for several reasons. The literature for microbial pathogenesis in plants lags at least 2 decades behind that of mammals. We have documented fewer than 300 SoCs from plant pathogens (viral, bacterial, and fungal). Our terminology for functions of SoCs from plant pathogens needs supplementation/improvement. Why? Because the innate immune system of plants, while at least as complex as that of mammals, is substantially different. The goals and ‘principles’ of immune defense are the same, but the individual cases and host molecules effecting the defensive effort are distinct. We are not as familiar with them. We plan on improving our understanding and our annotation effort for plant pathogen SoCs.

As the NSABB reviews the conduct of dual-use research oversight, it should consider how to incorporate our growing knowledge of SoCs into the biorisk management regime to ensure that life sciences research is conducted safely, securely, and responsibly. In the following section, we suggest several ways in which SoCs could be used to guide biorisk management decisions.

## How might annotated SoCs guide biorisk management decisions?

Since neither the DURC policies nor P3CO Framework provide guidance for scientists to judge whether proposed research is “reasonably anticipated” to result in a modified microbe with enhanced pathogenic properties, we propose leveraging our annotated SoCs as one indicator which could trigger greater scrutiny. We think our conception of SoCs provide clearer guidance than what currently exists. If the sequence being manipulated in the investigation is a direct-acting sequence of concern, and it is being expressed in an organism capable of causing disease in humans, then that work may require a higher level of oversight. This will depend on the likelihood that the resulting manipulated microbe poses a greater risk of infection or transmission than the unmodified microbe. So how might that risk be better adjudicated using SoCs annotated with FunSoCs and PathGO terms?

### Application to the USG DURC policy

Among the experiments of concern listed in USG DURC policy (Table 2), our conception of SoCs and their functions (FunSoCs) can help illuminate potential risks. Research that involves any one of three research activities: (i) transfer of a SoC to a different pathogen, (ii) alteration of a SoC such that the existing abilities of the original pathogen might be enhanced, or (iii) transfer of an SoC to a nonpathogenic microbe would trigger oversight. Our reflections on how FunSoCs might be used to better understand DURC follow and are summarized in Table 6.

**TABLE 6:** How work on SoCs could correlate with DURC categories.

Function of Sequence of Concern (FunSoC)	Case 1, SoC transferred to other pathogen or Case 2, SoC altered for enhancement of original pathogen	Case 3, SoC transferred to nonpathogen
Damaging	Could enhance the harmful consequences of the agent;	Might enable the nonpathogen to have harmful consequences
Immune Subverting	Enhances the harmful consequences of the agent; Disrupts immunity or the effectiveness of an immunization against the agent; Alters the host range or tropism of the agent; Enhances the susceptibility of a host population to the agent;	Might enable the nonpathogen to have harmful consequences; Might enable the nonpathogen to infect novel hosts; Might enhance the susceptibility of a host population to the agent;
Attachment Protein/Adhesin	Alters the host range or tropism of the agent; Enhances the susceptibility of a host population to the agent;	*Probably none*
Fusion Protein/Invasin	Alters the host range or tropism of the agent; Enhances the susceptibility of a host population to the agent;	*Probably none*
Dissemination	Enhances the harmful consequences of the agent; Increases the transmissibility or the ability to disseminate the agent; Enhances the susceptibility of a host population to the agent;	*Probably none*

**Damaging SoCs**: We briefly detailed four categories of damaging SoCs in our previous work (Godbold et al., 2022) and recapitulated them above. Inserting damaging SoCs into a microbe could violate Table 2 as it would be expected to enhance the harmful consequence of the agent. Such a result might also follow the alteration of a damaging SoC in its native microbe.

**Immune subverting SoCs**: As we discuss above, SoCs that subvert innate immunity may be more consequential than damaging SoCs. Results of experiments involving addition of these sequences to other microbes as well as modifications that might enhance their immune subverting abilities are also the most difficult to anticipate prior to the experiment. Such could “disrupt immunity against the agent” [Table 2] or “enhance the susceptibility of a host population to the agent” [Table 2]. It could also “increase the harmful consequence of the agent” [Table 2]. Alterations in some poxviral immune-evading sequences can change the host tropism of the virus [Table 2] (Bratke et al., 2013; Rahman and McFadden, 2017; 2020). Of course, these modifications depend on the experimental system and could very well be allowed after review. The study of immune subverting mechanisms of microbes in experimental infections of host organisms has produced numerous and important breakthroughs in our understanding of immunity.

**Adhesins** and **Invasins**: Adhesive properties are particularly abundant in biology. Adhesins are the molecules which primarily condition what cell types and what taxa are targeted by an infectious agent. As a result, their transplantation into a new organism might enable a change in host tropism [Table 2] or enhance the susceptibility of a new host population [Table 2]. Likewise, alterations of adhesins with the intention of altering host cell tropism should trigger a review. For viruses and for many other infectious agents that have an intracellular life cycle, the principal attachment protein (adhesin) is also responsible for viral fusion and subsequent cellular invasion. But there are dozens of bacterial invasins, not also adhesins, which manipulate extracellular matrix molecules or the cytoskeleton thereby leading to invasion. It is conceivable that altering these within a pathogen could lead to changes in host tropism [Table 2] or enhance the susceptibility of a new host population [Table 2]. Expressing adhesins/invasins from pathogens in nonpathogenic species will not generally violate DURC rules as they do not, by themselves, make a nonpathogenic microbe pathogenic (Schubert et al., 2004; Uchiyama et al., 2006; Pisano et al., 2012; Schmidgen et al., 2014).

**Dissemination factors**: There are disparate modes of action for dissemination factors, but the effect is that the infectious agent can spread within the host organism beyond what would be possible in the absence of the dissemination factor. This often occurs through the temporary subversion of host barriers. Addition of a foreign dissemination factor to an existing pathogen could lead to consequences that could increase the harmful effects of the agent [Table 2], increase the ability of the agent to disseminate in the host [Table 2], or even enhance the susceptibility of a host population to the agent [Table 2]. Modifications to a dissemination factor could conceivably affect each of these as well. Expression of a dissemination factor in a nonpathogen would be unlikely to make it pathogenic.

Of course, additions or alterations of SoCs to study the mechanism(s) would be less risky if performed in a microbe that was either not competent to replicate or otherwise incapable of causing human infection. If a SoC-based biosecurity regime were adopted, development of safer systems to study SoC function should be a focus of funding agencies.

## Implications for biosafety, biosecurity, and dual-use research oversight

As the global biorisk landscape evolves, it is necessary to update biorisk management policies and practices. As the NIH and OSTP reviews US dual-use research oversight policy, we think our approach to categorizing the functions of SoCs based on the published literature and using these as an aid for considering outcomes of organismal manipulation is a valuable addition and will strengthen existing policy. The rubric provided in Table 6, which maps the functions of SoCs onto different classes of experiments to suggest which DURC categories might be involved, could be helpful for considering the consequences of microbial modifications.

The United States has not provided any guidance for how to judge when the standard of “reasonably anticipated,” as used by the DURC policies and P3CO Framework, is met. This lack of detail and ambiguous terminology can be confusing for both researchers submitting proposals as well as scientists and funding agency officials involved in the peer review process. Therefore, NIH and OSTP should consider recommending that inserting or modifying SoCs with certain functions could be “reasonably anticipated” to lead to an enhanced phenotype covered by either set of policies. While this rule of thumb would not be the only determinant of whether an experiment was covered by DURC, it would increase the likelihood that potentially concerning research is subject to review under the appropriate policy. This approach will be particularly useful if NIH and OSTP adopts the recommendations from the National Science Advisory Board for Biosecurity (NSABB) to expand the scope and coverage of the P3CO and DURC policies. For example, NSABB proposed reducing the threshold for oversight of experiments with potential pandemic pathogens from those that are reasonably anticipated to generate a highly virulent or transmissible pathogen to those likely to generate a moderately virulent or transmissible pathogen. NSABB also recommended that the scope of the DURC policy be expanded from Tier 1 Select Agents to all human, animal, and plant pathogens (National Science Advisory Board for Biosecurity, 2023). These recommendations, when taken together, will subject a much broader swathe of pathogen research subject to oversight, necessitating the development of tools that can aid researchers and review entities in determining if proposed experiments could be “reasonably anticipated” to generate enhanced pathogens that require the implementation of risk mitigation measures prior to or following the research.

A similar lacuna in guidance for researchers on how to identify potential dual-use research exists in other countries that exercise some degree of oversight of dual-use research such as Australia, Canada, and the United Kingdom. These countries might also benefit from adopting functional criteria (like FunSoCs) into their education and awareness-raising activities to help scientists identify potential dual-use research. In addition, funding agencies in these countries could use FunSoCs as part of their screening process for grant proposals to determine if the research poses any dual-use risks that require mitigation.

For microbes that are increasingly synthetic, having their constituent sequences drawn from an expanding set of organisms, a screening approach based on taxonomy is likely to be of decreasing utility. In such cases a standard list of ‘bad sequences’ should be helpful in determining what microbes are likely to be concerning. An accurate computational assessment of the infectiousness of a synthetic microbe is not currently possible nor is it likely to be in the next decade. We think our work and that of others can provide pointers for how such as assessment might be attempted (Gemler et al., 2022; Godbold et al., 2022). Our criteria for functions of sequences of concern were described in our earlier publication and are available to the scientific community. Here we offer them as a useful framework for assessing risk in the context of dual-use research of concern.

SoCs cannot replace taxonomic lists of ‘bad bugs’, particularly in the case of viruses pathogenic for humans, which must remain part of any policy framework. But the addition of SoCs categorized by functions necessary for pathogenesis provides a useful supplement to such lists. The transfer of such sequences and their modification in ways that can be reasonably anticipated to enhance their damaging, disseminating, adhesive, invasive, or immune subverting effects should be noted in research proposals. Such a list of SoCs might allow the de-regulation of thousands of sequences from bacterial and eukaryotic pathogens that are presently deemed controlled. As suggested in Table 4 for *Bacillus anthracis*, over 99.5% of its 5,800 sequences play no distinct role in pathogenesis. Documenting and regulating the sequences that enable pathogenesis in nonviral organisms make it easier for researchers to investigate, *without oversight*, the biology of the remaining (and overwhelming) majority.

The revised guidance on DNA synthesis screening issued by the Department of Health and Human Services is undertaking a shift from a pathogen-based to a sequence-based approach^8^. Under the previous guidance, DNA synthesis providers were only required to screen orders against the genomes of a list of regulated pathogens. Under the revised guidance, “sequences that contribute to toxicity or pathogenicity” are considered sequences of concern that are covered by the guidance even if these are not encoded by a regulated biological agent. The NIH and OSTP could explore the desirability and feasibility of applying the broadly defined “sequences of concern” by the new HHS guidance to DURC oversight.

How can we be sure that the sequences enabling pathogenesis for these disease-causing microbes have been sufficiently investigated to find them all? This is something we cannot know, though there has been a great deal of work on most of the microbes found on select agent lists. Investing in research on the less well-investigated pathogens would help ensure that the most important pathogenic sequences are characterized. In addition, knowledge of the commonalities of sequences enabling pathogenesis that are a consequence of categorizing them might drive development of pathogen-agnostic therapeutics that may be able to neutralize widely shared mechanisms of pathogenesis.

One strategy to mitigate the risk of research involving SoCs is for funding authorities to encourage researchers to develop more, and more suitable, nonpathogenic microbial chassis to support the safe discovery of SoC functions. Once approved, these chassis could be used with decreased oversight. The use of non-replicating pseudoviruses can also be encouraged as a safer alternative to the insertion or modification of SoCs in pathogenic, replicating viruses.

A standardized and official list of SoCs with a set of approved annotations should be devised by governments whose scientists are involved in microbial pathogenesis research. How this list should be selected, maintained, and used is something that will need to be resolved. The process should involve consultation among experts in infectious diseases and policy as well as relevant biodefense professionals. The first question for such a group involves deciding which host taxa needing protection should be selected. Humans are the primary concern, but animals and plants that dominate a country’s agriculture should probably also be considered. Once the hosts are established, the pathogens that afflict these hosts can be determined. Then SoCs will be documented from this list of pathogens.

The availability of such a list and the type of information it should provide is also something to be decided. Should it be an open list of sequence names? A list of sequence names with accession numbers? The names, accession numbers, and a tabular list of problematic functions (i.e., damaging, immune subverting, adhesive, etc.)? Should terse but specific descriptions of pathogenic activity such as FunSoCs and PathGO be associated with each SoC? Or a more detailed description of how it interacts with host molecules? Should citations/references of the primary or secondary literature be required to justify the functional determinations for each sequence?

Who should have access to these lists? Should it be publicly available to the scientific community at large? Should only institutional review entities responsible for implementing DURC oversight of research conducted at their institution have access to this information? Should different groups have access to lists of differing comprehensiveness? The utility of such a tool for enhancing DURC oversight needs to be balanced with the information hazards presented by an accessible compilation of sequences that enable pathogenesis. Those making these decisions will be threading the needle to best serve the interests of public safety, open research, and international security.

## Conclusion

Thoughtful researchers who work with pathogenic microbes are usually aware of the hazards involved in introducing changes into sequences involved in pathogenesis. We think SoCs annotated with FunSoCs will bring further clarity to help ascertain when more care should be taken in experiments, especially in fully replication-competent organisms. We believe that SoCs can be a useful component of the regulatory regime that governs sequences acceptable for insertion and alteration in pathogenic agents. We have delineated bioengineering situations that could be ‘reasonably anticipated’ to improve the disease-causing capacity of pathogens. We believe that these guidelines have the potential to reduce the risk of accidentally generating an ‘improved’ pathogen while promoting awareness of the phenotype effects of potentially concerning genotypic changes. We think considering SoCs by function improves the probability that potentially concerning research is subject to the appropriate level of oversight to ensure that such research is conducted safely, securely, and responsibly.

## Footnotes

1
https://www.grassley.senate.gov/imo/media/doc/national_institutes_of_health_to_grassley_-_covid_origins_grant_oversight.pdf.

2
https://theintercept.com/2021/09/09/covid-origins-gain-of-function-research/and https://theintercept.com/2021/10/21/virus-mers-wuhan-experiments/.

3
https://theintercept.com/2021/09/23/coronavirus-research-grant-darpa/.

4
https://cms.wellcome.org/sites/default/files/wtp059491.pdf.

5
https://www.canada.ca/en/public-health/programs/consultation-biosafety-guideline-dual-use-life-science-research/document.html.

6
https://www.nhmrc.gov.au/file/18130/download?token=anGdkE4f.

7
https://github.com/jhuapl-bio/pathogenesis-gene-ontology.

8
https://aspr.hhs.gov/legal/syndna/Pages/default.aspx.
